# Efficient Synthesis and Characterization of Polyaniline@Aluminium–Succinate Metal-Organic Frameworks Nanocomposite and Its Application for Zn(II) Ion Sensing

**DOI:** 10.3390/polym13193383

**Published:** 2021-09-30

**Authors:** Amjad E. Alsafrani, Waheed A. Adeosun, Hadi M. Marwani, Imran Khan, Mohammad Jawaid, Abdullah M. Asiri, Anish Khan

**Affiliations:** 1 Chemistry Department, Faculty of Science, King Abdulaziz University, P.O. Box 80203, Jeddah 21589, Saudi Arabia; Amjad.chem@hotmail.com (A.E.A.); dsnwaheed1@gmail.com (W.A.A.); aasiri2@kau.edu.sa (A.M.A.); 2 Department of Chemistry, College of Science, University of Jeddah, Jeddah 21589, Saudi Arabia; 3 Center of Excellence for Advanced Materials Research, King Abdulaziz University, P.O. Box 80203, Jeddah 21589, Saudi Arabia; 4 Applied Sciences and Humanities Section, Faculty of Engineering and Technology, University Polytechnic, Aligarh Muslim University, Aligarh 202002, India; imrannano@gmail.com; 5 Laboratory of Biocomposite Technology, Institute of Tropical Forestry and Forest Products (INTROP), University Putra Malaysia (UPM), Serdang 43400, Selangor, Malaysia

**Keywords:** PANI@Al-SA modified electrode, MOFs, composite, linear sweep voltammetry

## Abstract

A new class of conductive metal-organic framework (MOF), polyaniline- aluminum succinate (PANI@Al-SA) nanocomposite was prepared by oxidative polymerization of aniline monomer using potassium persulfate as an oxidant. Several analytical techniques such as FTIR, FE-SEM, EDX, XRD, XPS and TGA-DTA were utilized to characterize the obtained MOFs nanocomposite. DC electrical conductivity of polymer-MOFs was determined by four probe method. A bare glassy carbon electrode (GCE) was modified by nafion/PANI@Al-SA, and examined for Zn (II) ion detection. Modified electrode showed improved efficiency by 91.9%. The modified electrode (PANI@Al-SA/nafion/GCE) exhibited good catalytic property and highly selectivity towards Zn(II) ion. A linear dynamic range of 2.8–228.6 µM was obtained with detection limit of LOD 0.59 µM and excellent sensitivity of 7.14 µA µM^−1^ cm^−2^. The designed procedure for Zn (II) ion detection in real sample exhibited good stability in terms of repeatability, reproducibility and not affected by likely interferents. Therefore, the developed procedure is promising for quantification of Zn(II) ion in real samples.

## 1. Introduction

Metal-organic frameworks (MOFs) have attracted great interests in the recent years as it is a class of hybrid nanoporous materials with crystalline architectures linked by metal ions (or clusters) coordinated with multifunctional organic linkers [1]. Owing to their unique and advantageous properties such as high specific surface area and permanent porosity, tunable sizes, and controllable functionality, MOFs have been widely utilized in numerous fields of applications such as energy conversation, storage applications, chemical sensing, renewable energy, and separation [2,3,4,5]. These MOFs materials are suitable for applications as electrochemical sensing as well as surface modifiers because of their high surface area and pore volume, good absorbability, and high catalytic activity [6,7]. However, most MOFs still have poor electrical conductivities due to the insulating character of the organic ligand, resulting in limited applications in electrochemical sensors [6,8].

For this reason, few research studies are obtainable regarding the employability of MOFs as modifiers of electrode surfaces for application as electroanalytical sensors [9]. Therefore, the key for improving sensing performance is to combine MOFs with functional materials that have high redox activity and electrical conductivity such as, electronically conducting polymers [6,10,11,12].

Among electronically conducting polymers, particular polypyrrole(PPy), polythiophene (PTh) and polyaniline (PANI) have received considerable attention. PANI exhibits reasonable potential as the electrode material in electrochemical sensing filed, because of its high electrical conductivity in partially oxidized state, high theoretical capacitance, good thermal conductivity, simple fabrication and excellent environmental stability [13].

Zeinab et al. [14] showed that PANI/Cu-MOF composite had an effective method to improve the electrochemical properties, including cycle life and the specific capacitance of Cu-MOF. The data of Konstantinet al. [12] pointed out that introduction of Fe-BTC MOF on PANI-based composite enhanced the electrochemical response of the material. Chunme et al. [15] fabricated hierarchical ZnO@MOF@PANI core-shell nanoarrays on carbon cloth for SC electrodes, demonstrating an ultrahigh capacitance that reaches up to 340.7 F·g^−1^ of 1A·g^−1^ with a good rate capability.

Cations are essential in a wide variety of processes such as, medical, biological and chemical as well as contributing significantly to environmental pollution. Among the various cations, Zn(II) ion is detecting in wastewater coming from paper-manufacturers, paints and pigments, pesticide, and even pharmaceuticals, which can cause negative effects on human health [15]. Therefore, it is necessary to punctually detect Zn(II) ions concentration by architecting an electrochemical sensor.

**Scheme 1:**
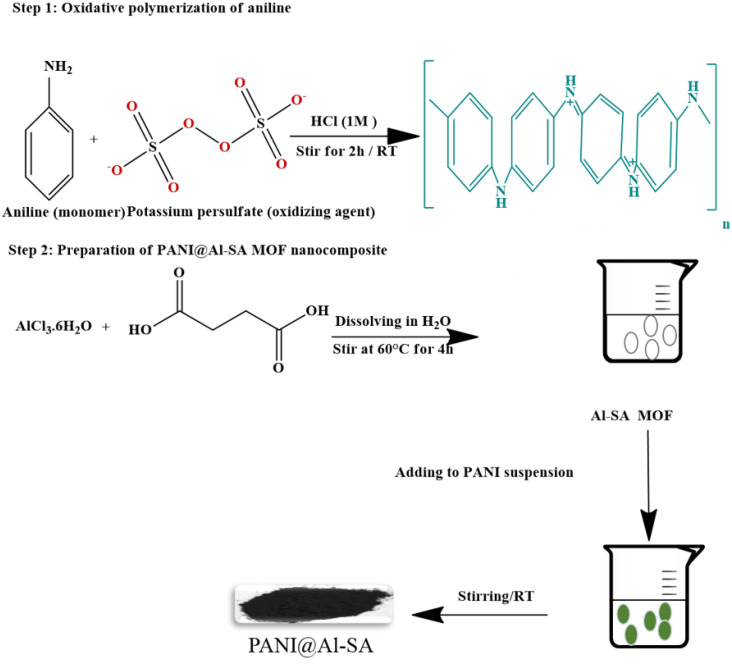
Schematic representation for the synthesis of PANI@Al-SA MOFs nanocomposite.

In the present study, an electrochemical sensor aluminum–succinicate MOF based on the incorporation of conducting polyaniline was synthesized as shown in Scheme 1 and explored the electrical properties. PANI@Al-SA MOF nanocomposite employed as modifier for glassy carbon electrode (GCE). Electrochemical sensing performance of the proposed sensor was investigated using cyclic voltammetric, impedance spectroscopy and linear sweep voltammetry techniques at room conditions.

## 2. Experimental

### 2.1. Reagents and Instruments

All reagents used were of high purity and of analytical reagent grade. Doubly distilled water (DDW) was used throughout the all experiments. Distillation water (18.6 MΩ·cm^−1^) was prepared by a Millipore Milli-Q Plus Ultra-Pure Water Purifier (Millipore, Bedford, MA, USA) and all solutions were prepared in the standard method by dilution as with distilled water. Aniline (C6H_5_NH_2_), hydrochloric acid (HCl), potassium persulfate (K_2_S_2_O_8_), aluminum chloride hexahydrate (AlCl_3_-6H_2_O), succinic acid (C_4_H_6_O_4_), sodium hydroxide (NaOH), potassium ferricyanide (K_3_[Fe(CN)_6_]), zinc sulfate (ZnSO_4_), ethanol (C_2_H_5_OH), 5% nafion (ethanolic solution), potassium dihydrogen phosphate (KH_2_PO_4_), dipotassium hydrogen phosphate (K_2_HPO_4_) and all other chemicals were purchased from Sigma-Aldrich (St. Louis, MO, USA).

Following instruments were used during present research study: A Perkin Elmer Spectrum 100 series FT-IR spectrometer (Beaconsfield, Bucks, UK), a field emission-scanning electron microscope (FESEM) JEOL J SM 7600F, Japan was used that was linked with an Oxford-Isis (UK) X-ray microanalysis system (EDX), a Barnstead Thermolyne 47900 benchtop muffle furnace (Waltham, MA, USA). A WTW model 7200 laboratory pH meter (inoLab, Weilheim, Germany) was used for the pH measurements, The X-ray photoelectron spectroscopy (XPS, PHI5000 VersaProbeII, ULVAC-PHI Inc, Kanagawa, Japan) and the X-ray diffraction spectroscopy (XRD) analysis were carried out using Thermo scientific diffractometer with a Cu Kα radiation source (λ = 0.15405 nm). The thermal decompositions were carried out via simultaneous TGA and DTA with a Mettler Toledo TGA/DSC 1 STARe thermal analyzer (Mettler Toledo, Greifensee, Switzerland), between 50 to 900 °C at a heating rate (20 °C min^−1^) in the nitrogen atmosphere (nitrogen flow rate of 20 mL min^−1^). The electrochemical analysis was performed using Electrometer (Keithley, 6517A, AccuSource Electronics, Inc, Gainesville, FL, USA) and Autolab potentiostat (PGSTAT302N-AUT85887) powered by Nova1.10 software. The electrodes used in the study are reference electrode (Ag/AgCl), auxiliary electrode (Pt wire, 0.5 mm in diameter) and working electrode (GCE, 0.07 cm^2^).

### 2.2. Preparation of PANI@Al-SA MOFs Nanocomposite

PANI@Al-SA MOFs nanocomposite was synthesized in three steps: firstly, Al-SA MOF was synthesized according to the procedure in the literature with slight modification [5]. 6.34 g of AlCl_3_-6H_2_O was dispersed in100 mL of DDW in a 250 mL conical flask and stirred at 60 °C for 2 h. In a 250 mL separate conical flask, 6.2 g of succinic acid was mixed with 4.75 g of NaOH (0.5:1:2.2, Al:SA:NaOH) and dispersed in 100 mL DDW with continues stirring until completely dissolved. After that the precursor containing succinic acid was slowly added into the mixture containing aluminum precursor, where a white suspension was produced after mixing. The mixture was then continuously stirred at 60 °C for 4 h. The obtained white suspension was kept overnight at room temperature.

The oxidative polymerization of the monomer aniline was achieved by adding the different volumes percent (7% and 10%) of aniline prepared in 0.1 M HCl in 200 mL 0.1 molar potassium persulphate under continuous stirred at room temperature for 2 h. The obtained green gel of polyaniline was cooled in the refrigerator and digested overnight below 5 °C for complete polymerization.

Finally, the gel of polyaniline was added to the white suspension of Al-SA and stirred at room temperature until the color of mixture turned into blackish slurries. This was then put at lab temperature for overnight and then was filtered off, washed with DDW several times until free form impurities and dried at 50 °C overnight in an oven.

### 2.3. Electrical Conductivity Measurements

For doping, PANI@Al-SA nanocomposite was treated with 1.0 M aqueous HCl, then washed with distilled water to remove any residual acid. Then, it was dried in an oven overnight at 50 °C. 300 mg portions of the nanocomposite were grained by a mortar and pestle then the sample was transfers by spatula into a die of definite dimensions. That die was put under the hydraulic pressure machine for 25 min at 25 kN, and a screw gauge was used to measure the thickness of the pellets. A DC electrical conductivity measuring instrument machine (SES Instrument Pvt. Ltd., Roorkee, India), was used for measuring of the conductivity of the pressed pellet at room temperature by using a four probe method for semiconductors. This is the most popular technique used in case of conductivity measurement for semiconductor materials instead of the classical technique (i.e., two probes). For determination of electrical conductivity, the current—voltage data recorded at room temperature were fitted by the following Equations (1)–(4) [16]:

(1) $$\rho =\rho_{{}^{\circ}}/G_{7}\left(\frac{W}{S}\right)$$

In this relationship ρ denotes the corrected resistivity (Ω•cm), $\rho_{{}^{\circ}}\;$ denotes to uncorrected resistivity and $G_{7}\left(\frac{W}{S}\right)$ is shown the correction factor used in case of non-conucting bottom surface. The W and S symbols refer to the thickness of the pellet (cm) and the probe spacing (cm), respectively.

The $G_{7}\left(\frac{W}{S}\right)$ and $\rho_{{}^{\circ}}$ can be obtained from the following equations:

(2) $$G_{7}\left(\frac{W}{S}\right)=\left(\frac{2S}{W}\right)ln2$$

(3) $$\rho_{{}^{\circ}}=\frac{V}{I}\times 2\pi S$$

Then, the electrical conductivity σ is calculated using the equation:

(4) $$\sigma =\frac{1}{\rho }$$

where I refer to the current in Ampere, *V* refer to the voltage in Volt and σ is the electrical conductivity (S•cm^−1^).

### 2.4. Electrochemical Sensor Fabrication

Deionized water–ethanol solution and 0.3 μm alumina slurry were used to clean and polish glassy carbon electrode, respectively. 5 mg of PANI@Al-SA nanocomposite was mixed in 20.0 μL ethanolic solution and sonicated for 5 min. Subsequently, 10 μL of the homogenized suspension was dispersion onto the surface of GCE with addition of a drop of 5% ethanolic Nafion. The modified GCE was then allowed to dry for 3 h at room temperature.

### 2.5. Electrochemical Measurements

The electron transfer rate was evaluated in 0.1 mM [Fe(CN)_6_] ^3−/4−^/0.1 M KCl by cyclic voltammetry (scan rate of 100 mV/s, scanning potential of −0.1 V to 1 V and step potential of 0.0082 V).

EIS study was carried out in 0.1 mM [Fe(CN)_6_] ^3−/4−^ in 0.1 M KCl at a frequency range from 0.0001 KHz to 0.1 KHz and a DC potential of +0.35 V.

CV and LSV methods were used to detect zinc ion, with a scan rate of 100 mV/s, potential, scanning potential of −1.6 V to 1 V and 0.0082 V step potential.

Response time of Zn(II) sensor was evaluated in the solution containing 20 μM Zn(II) in 0.1 M acetate buffer of pH 4.5 by using Electrometer.

### 2.6. Real Sample Analsis

Real water samples, including bottled water and tap water were subjected to the determination of Zn(II). Bottled water was purchased from local market. Tap water sample was attained from our research laboratory (King Abdulaziz University, Jeddah, Saudi Arabia). A 2 mL of water samples was diluted to 25 mL of 0.1 M acetate buffer medium of pH 4.5 as supporting electrolyte. After spiked known concentration of Zn(II) ions, the recovery studies of spiked was carried.

## 3. Results and Discussion

### 3.1. Physiochemical Characterization of PANI@Al-SA Nanocomposite

#### 3.1.1. Fourier Transform Infra-Red (FT-IR) Analysis

**Figure 1:**
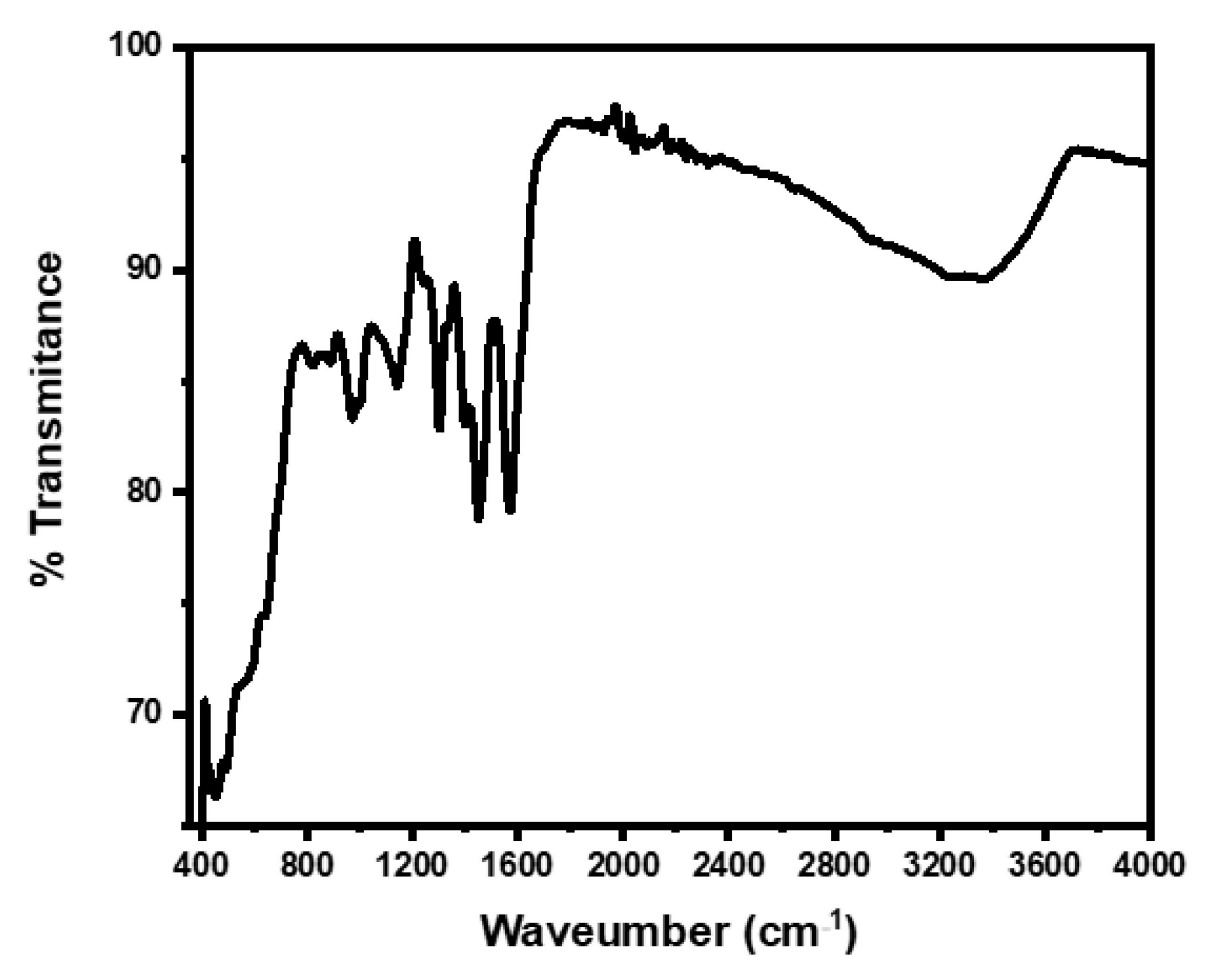
Fourier Transform Infra-Red (FT-IR) analysis spectrum of PANI@Al-SA MOFs nanocomposite.

The structure of PANI@Al-SA was studied using FT-IR spectroscopy. The FT-IR spectrum of PANI@Al-SA nanocomposite as shown in Figure 1, strong peaks at 1567 and 1448 cm^−1^ corresponding to the C=C stretching vibration of the quinonoid and benzenoid rings, respectively, indicating that the prepared polyaniline is in its conducting emeraldine state [17]. A big-intensity peak appeared in the range of 3200–3400 cm^−1^ is attributed to the N–H bond stretching vibration. The Al-SA MOF show nearly identical numbers locations of the IR peaks in the region of 600–3100 cm^−1^. The stretching vibrations of the sp3 C-H arising from α-hydrogens of succinic acid appear near 2907 cm^−1^. Both stretching vibrations asymmetric and symmetric of the COO- can be recognized nearly in a similar range at 1550–1100 and 1429 cm^−1^, respectively. In addition, multiple peaks appeared within 400–1100 cm^−1^ region are caused by a unique characteristic of Al-O and Al-O-H vibrations in an octahedral coordination state [5].

#### 3.1.2. Morphological and Elemental Analysis

**Figure 2:**
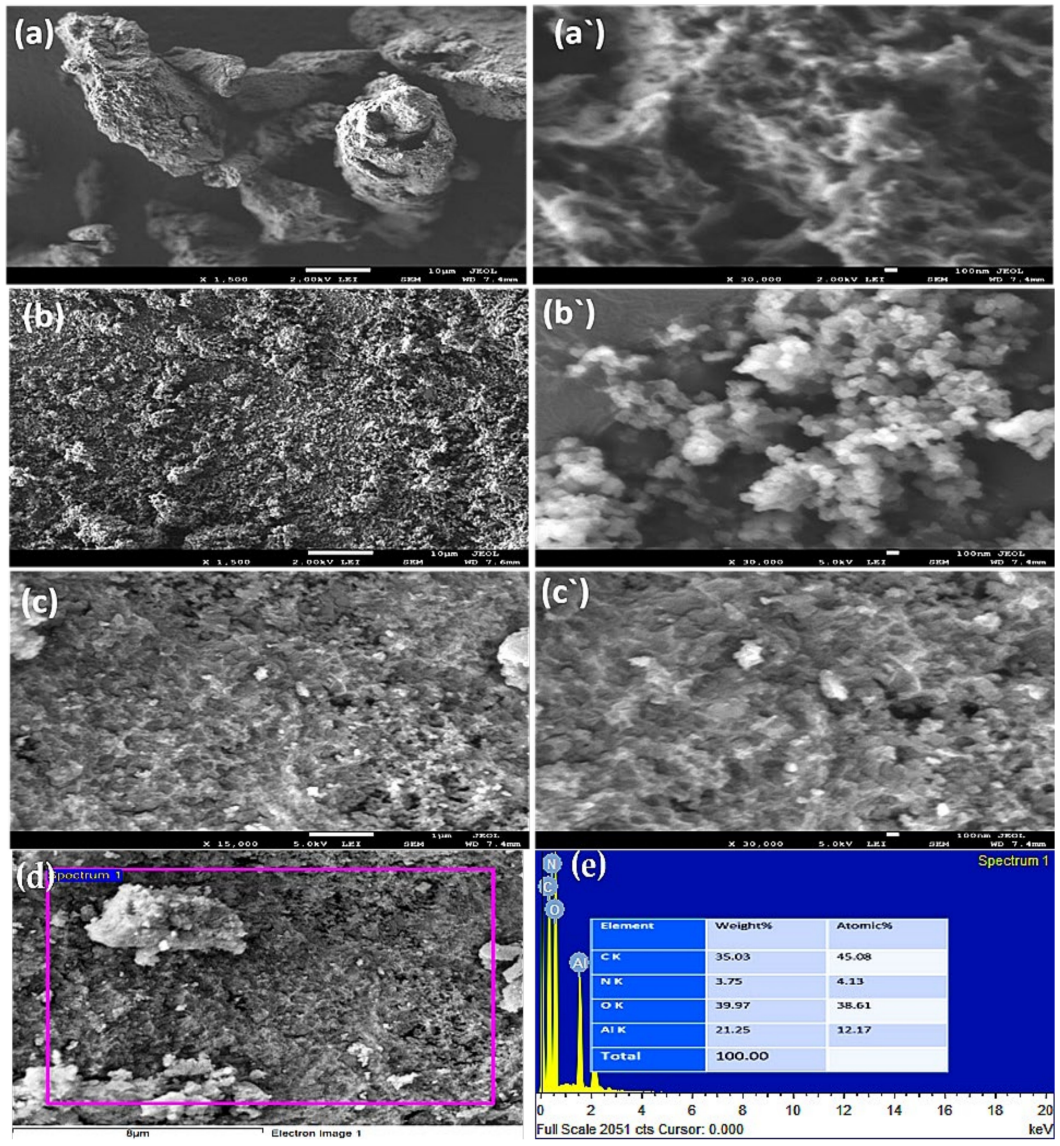
FE-SEM images of (**a,a^˴^**) PANI, (**b,b^˴^**) Al-SA and (**c,c^˴^**) PANI@Al-SA at different magnification (low and high magnified images) and EDX spectrum of (**d**,**e**) PANI@Al-SA. **(a’, b’, and c’ denote higher magnification).

For investigation the morphologies of PANI, Al-SA and PANI@Al-SA nanocomposite, Field Emission Scanning Electron Microscopy (FE-SEM) was used for taking images at different magnification (Figure 2). Figure 2c,c’ demonstrates the clear differences with the formation of organic polymer-MOF nanocomposite after adhesion PANI into MOF matrices. Also, it displays thicker and denser for some nanoparticles at higher magnification (Figure 2c’) more than Al-SA MOF (Figure 2b’). Furthermore, the EDX given a quantitative and qualitative information related to the elemental composition of material. The PANI@Al-SA is composed of oxygen (35.03%), nitrogen (3.75%), oxygen (39.97) and aluminum (21.25%) as obtained from EDX spectrum in Figure 2d,e.

#### 3.1.3. X-ray Photoelectron Spectroscopy (XPS)

**Figure 3:**
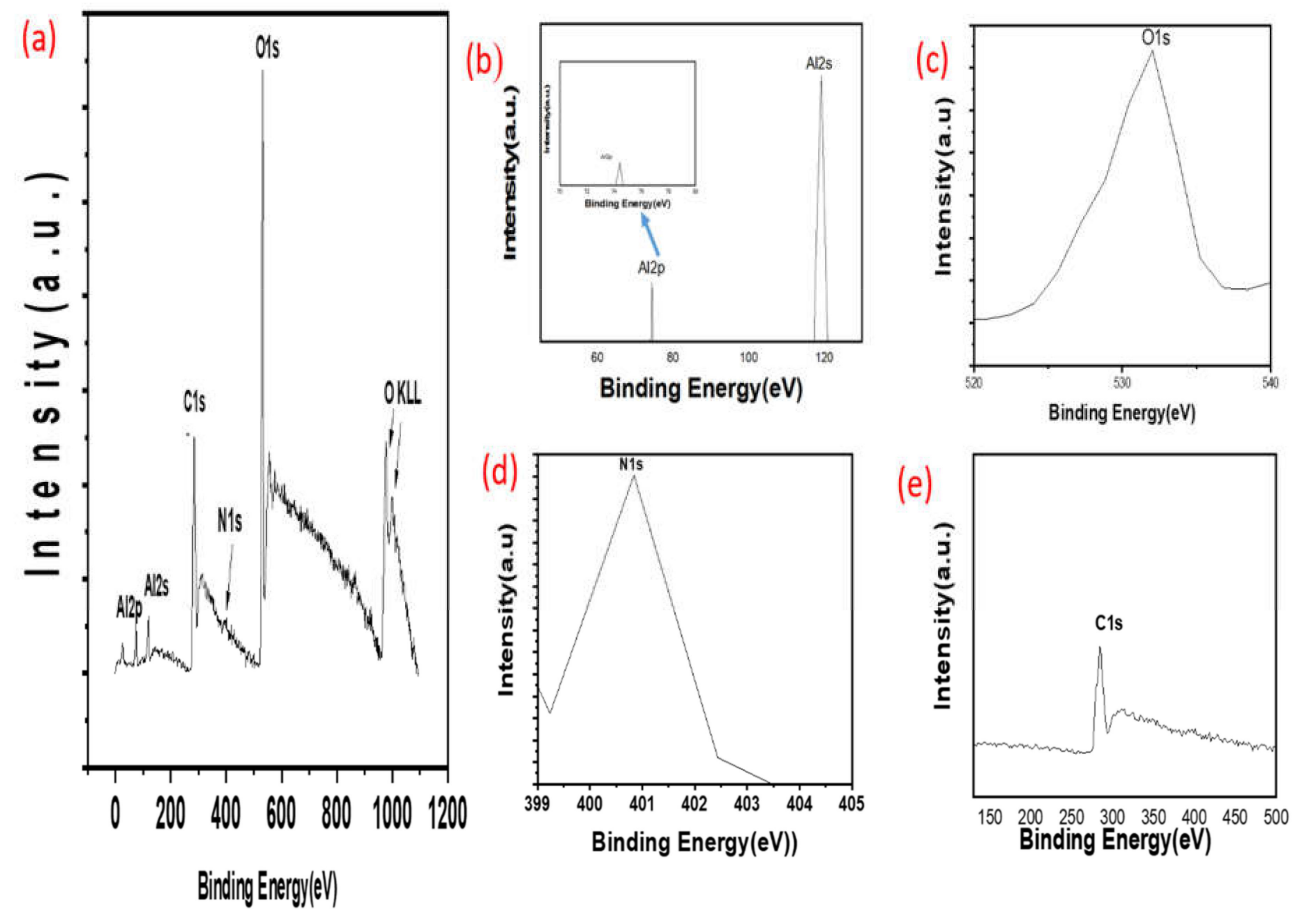
XPS spectrum showing binding energy for (**a**) PANI@Al-SA, (**b**) Aluminum, (**c**) Oxygen, (**d**) Nitrogen and (**e**) Carbon.

To complete SEM imaging and other characterization techniques with data regarding the chemical composition of the PANI@Al-SA nanocomposite, XPS analyses were used for PANI@Al-SA nanocomposite to examine the chemical or electronic states of oxygen, carbon, aluminum and nitrogen. Figure 3a showed the full spectrum of the nanocomposite PANI@Al-SA. The binding energy appeared at 532 eV corresponds to O1s (Figure 3b). For the C1s spectrum as presented in Figure 3c, the binding energy appears around 285.6 eV for the covalent bond, equivalent to C-C chain existed in PANI skeleton presence in PANI@Al-SA nanocomposite [18,19]. The peak at 400.8 eV in Figure 3d is ascribed to N1s in the PANI@Al-SA nanocomposite. The spectrum of Al is shown in Figure 3e, can also be equipped with two fine peaks Al 2s and Al 2p are located at 119 eV and 74.7 eV, respectively. Therefore, it can be assumed that the PANI@Al-SA nanocomposites contained four precious elements.

#### 3.1.4. X-ray Diffraction (XRD)

**Figure 4:**
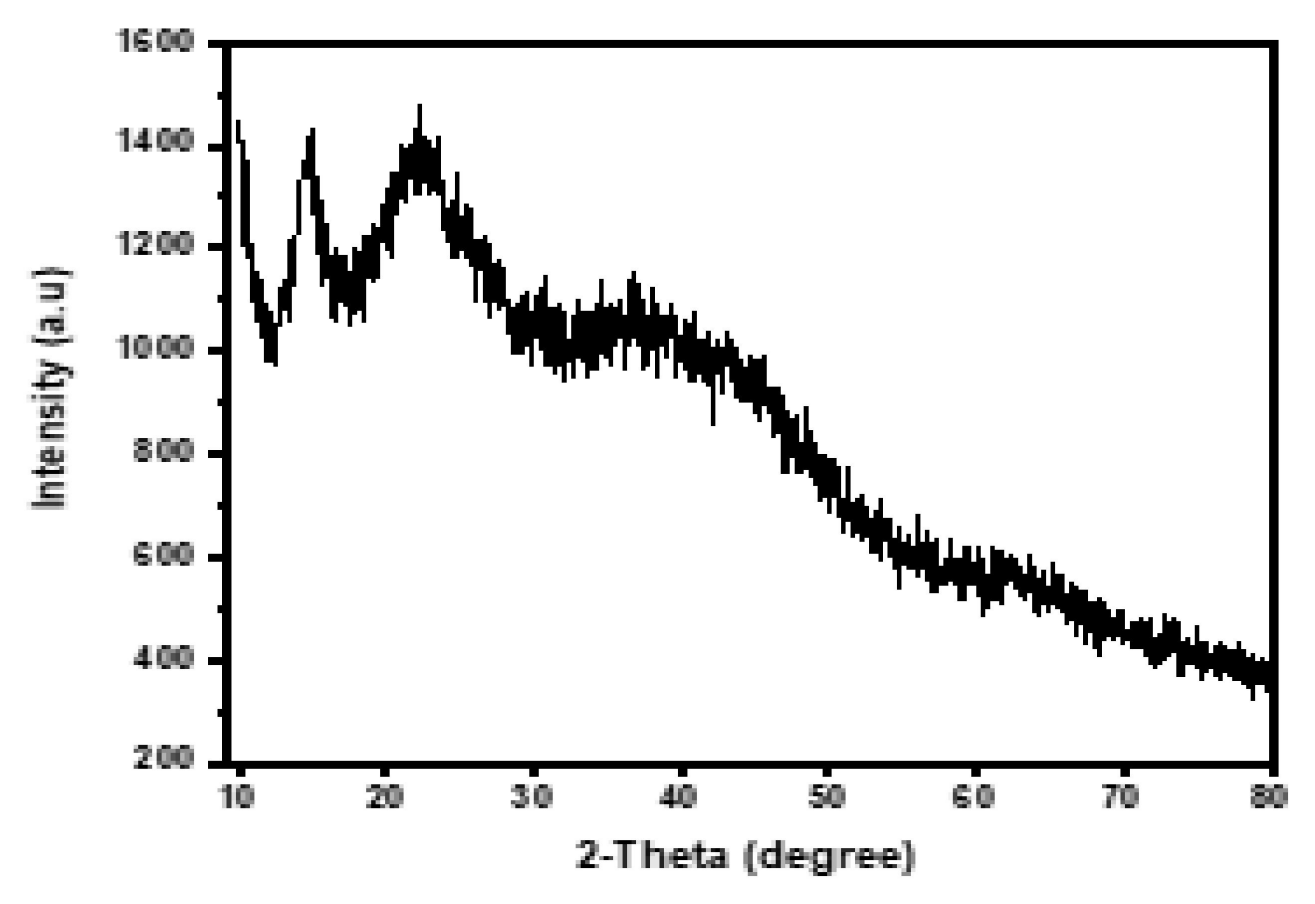
Powder XRD patterns for the PANI@Al-SA MOF nanocomposite.

X-ray powder diffraction was used to investigate the crystal structure of PANI@Al-SA nanocomposite. Figure 4 shows two peaks with 2θ values at 14.77 and 22.64. The broadening of these diffraction peaks for PANI@Al-SA nanocomposite can be attributed to the small crystal grains and corresponds to the amorphous nature of polyaniline composite [20].

#### 3.1.5. Thermogravimetric Analysis—Differential Thermal Analysis (TGA-DTA)

**Figure 5:**
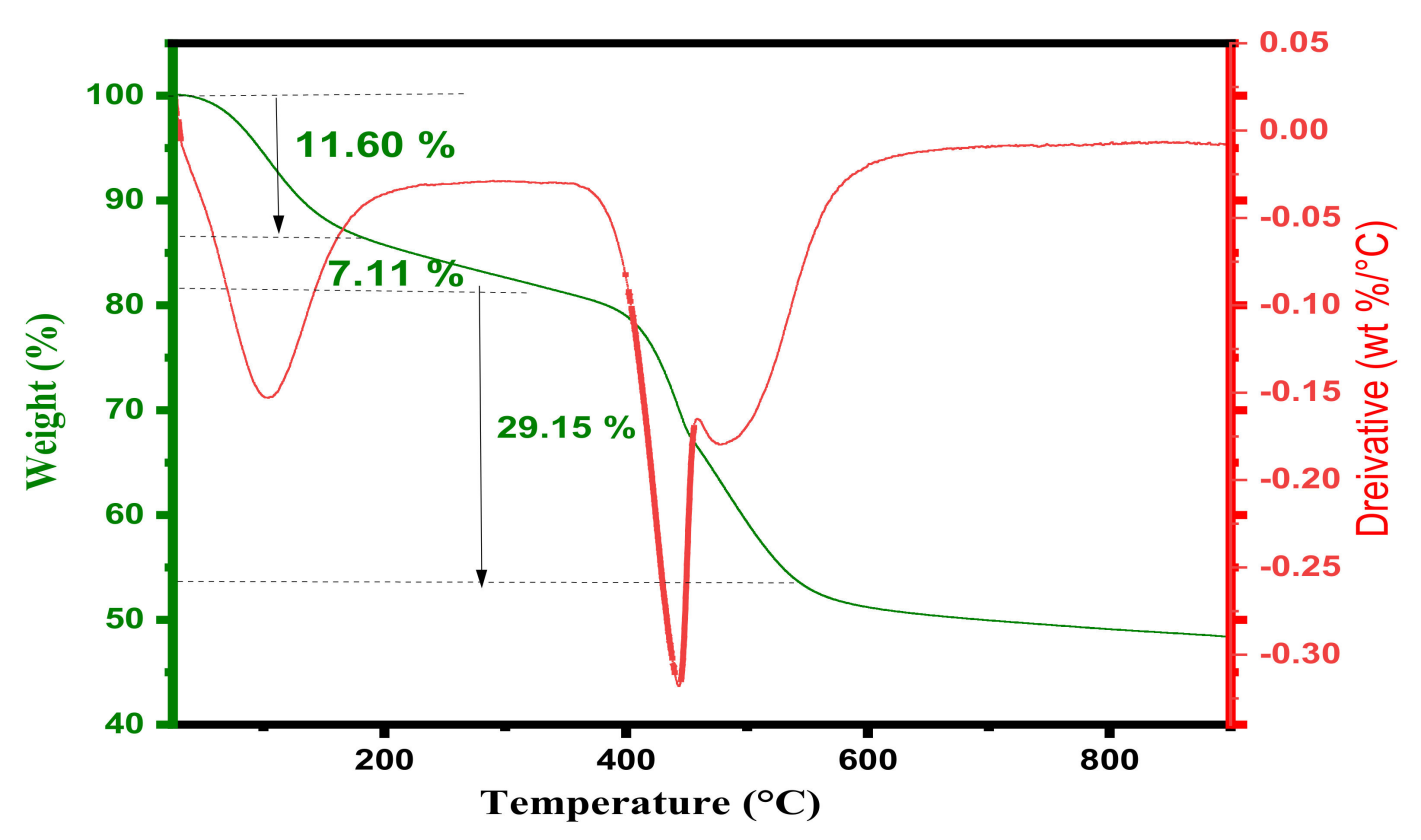
Parallel TGA-DTA curves of PANI@Al-SA.

To obtain more information about the thermal behavior of PANI@Al-SA nanocomposite, thermogravimetric analyses (TGA-DTA) curves were obtained as presented in Figure 5. In the initial step, a weight loss of mass about 11.6% is observed in the temperature below 100 °C due to loss of surface water molecule. The weight loss noticed between 150–350 °C may be attributed to the decomposition of Al-SA MOF. A weight loss of mass about 29.15% has been observed at between 350 to 550 °C corresponds to decomposition or the thermal degradation accompanied with the crosslinking process in the polyaniline polymer matrix [21]. The DTA curve displays the reaction is endothermic during the change of phase of the nanocomposite, peaks observed at 101, 440 and 480 °C likely associated with dehydration and decomposition of the PANI@Al-SA nanocomposite.

#### 3.1.6. Electrical Conductivity Study of PANI@Al-SA

Conducting polymer, polyaniline is the main ingredient that makes the nanocomposite electrically conductive. The electrical conductivity of the composite is attributed to oxidized polyaniline maintained in its conductive state by polyaniline@Al-SA. The electrical conductivity σ of the PANI@Al-SA nanocomposite samples prepared with varying % vol concentration of PANI (7 and 10) at room temperature and its estimated under ambient conditions [22]. In addition, the composite materials treated in 0.1 M HCl due to the improvement the electrical conductivity by a the charge-transfer reaction between the composite’s polyaniline component and the agent HCl doping [23]. The electrical conductivity of the 10% PANI@Al-SA (4.394 × 10^−4^ S•cm^−1^) is higher than that obtained with 7% PANI@Al-SA (1.615 × 10^−8^ S•cm^−1^). Moreover, the electrical conductivity obtained with 10 % PANI@Al-SA is in the range of polyaniline composites (10^−5^ to 10^−3^ S•cm^−1^) [23]. By taking into consideration of this result, 10% PANI@Al-SA was employed for the modification of GCE to detect metal ions in aqueous solution in the further study.

### 3.2. Electrochemical Characterization of PANI@Al-SA Nanocomposite

#### 3.2.1. Cyclic Voltammetry Behavior of Bare and Modified Electrodes in [Fe(CN)_6_] ^3−/4^^−^ Solution

**Figure 6:**
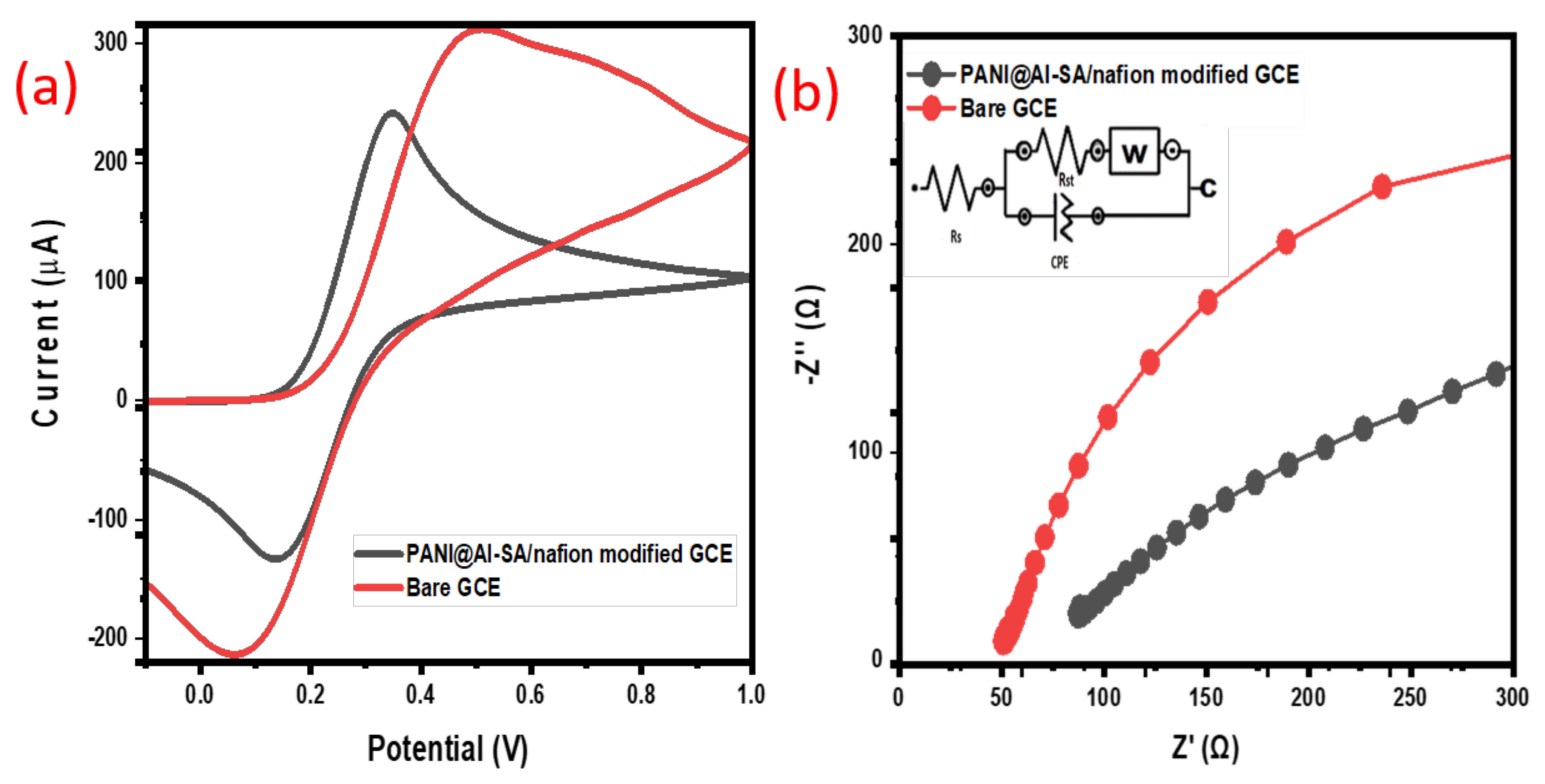
(**a**) Cyclic Voltammograms of 0.1 mM [Fe(CN)6] ^3−/4−^ in0.1M KCl at a scan rate of 100 mV/s, (**b**) Nyquist plot for bare and PANI@Al-SA/nafion modified GCEs in 0.1 mM [Fe(CN)6] ^3−/4−^/0.1 M KCl. The inset represents a Randel circuit diagram for the system where is Rs is the resistance of the solution, Rct is the resistance of the charge transfer, CPE is constant phase element and W is Warburg impedance.

The property of the electrode surface before and after deposition of conductive PANI@Al-SA/nafion on the GCE was carried out. The electron transfer rate was investigated by peak-to-peak separation potential ΔEp [24]. It can be evaluated by studying redox-reaction of 0.1 mM [Fe(CN)_6_] ^3−/4−^ in 0.1M KCl. The cyclic voltammogram shown in Figure 6a revealed the redox peak separation ΔEp for PANI@Al-SA modified GCE and bare GCE. It is clear from the figure that PANI@Al-SA/nafion modified had a smaller value (0.2 V) than bare GCE (0.4 V). This confirms that a conductive nanocomposite has been deposited on the surface of GCE.

Moreover, electrochemically effective surface area of (EESA) of PANI@Al-SA/nafion modified GCE was calculated according to the Randles–Sevcik equation [24]:

i_p_ = (2.69 × 10^5^) × A × n^3^^/2^ × D^1^^/2^ × C × v^1^^/2^ (5)

where, i_p_ is the peak current in Ampere, n is the number of electrons transferred (n=1),

A (cm^2^) is the effective surface area of the electrode, C is the concentration of the electroactive species in the electrolyte in mole/cm^3^, D (cm^2^ s^−1^) refer to the diffusion coefficient (for aqueous ferrocyanide is 7.6 × 10^−6^ cm^2^ s^−1^) and v is the scan rate in volt per second that was determined by cyclic voltammogram at various scan rate (5–50 mV/s), in 1mM ferricyanide couple (in 0.1 M KCl) as presented in Figure S1a. By combination, the Equation (5) and the slope of anodic peak (i_p_) current against square root of scan rate (v^1/2^) (Figure S1b), effective surface area PANI@Al-SA/nafion modified GCE was determined to be 0.86 cm^2^. By taking account the surface area of commercial bare electrode is 0.07 cm^2^, according to the manufacturer, the effective surface area was increased by 91.9% after deposition of conducting PANI@Al-SA film. The result obviously shows that PANI@Al-SA provide more binding sites owing to a large surface area of Al-SA MOF.

#### 3.2.2. Electrochemical Impedance Spectroscopy (EIS) Analysis

The current resistivity property of the material is estimated using the EIS approach. It is an effective technique for evaluating the rate of electron transport and interfacial surface conductivity of the PANI@Al-SA/nafion /ferricyanide solution. The impedance spectrum typically expressed as a Nyquist plot, which is a diagram that depicts the relationship between imaginary (Z”) and real (Z’) part of the impedance. It comprises a semi-circular diameter at higher frequency, namely charge transfer resistance (Rct) indicates to electron transfer limited process and a diagonal line at lower frequency denotes to the diffusion process. Figure 6b presents the Nyquist plot in terms of imaginary (Z”) and real (Z’) part of impedance. As shown in Figure 6b, a semicircle segment of bare GCE in 0.1mM [Fe(CN)_6_]^3−/4−^ with Rct of 1183.3 Ω (Figure S2a) was reduced to 612.82 Ω (Figure S2b) with that of PANI@Al-SA/nafion modified GCE. This improvement in value of Rct for PANI@Al-SA nanocompoite ascribed to the presence of PANI with high conductivity and better electron transport for this electrode. Thus, the improved electron transport indicated that the surface area was increased as well as conductance of PANI@Al-SA nanocomposite. Other parameters which are known as simplified Randle’s circuit related EIS data that obtained by fitting with assistance Nova 1.1 in—constructed EIS-fit software via non-linear least squares approaches were tabulated in Tables S1 and S2.

### 3.3. Electrochemical Sensing of Zn^2+^ by PANI@Al-SA Modified GCE

#### 3.3.1. Effect of pH as Supporting Electrolyte

**Figure 7:**
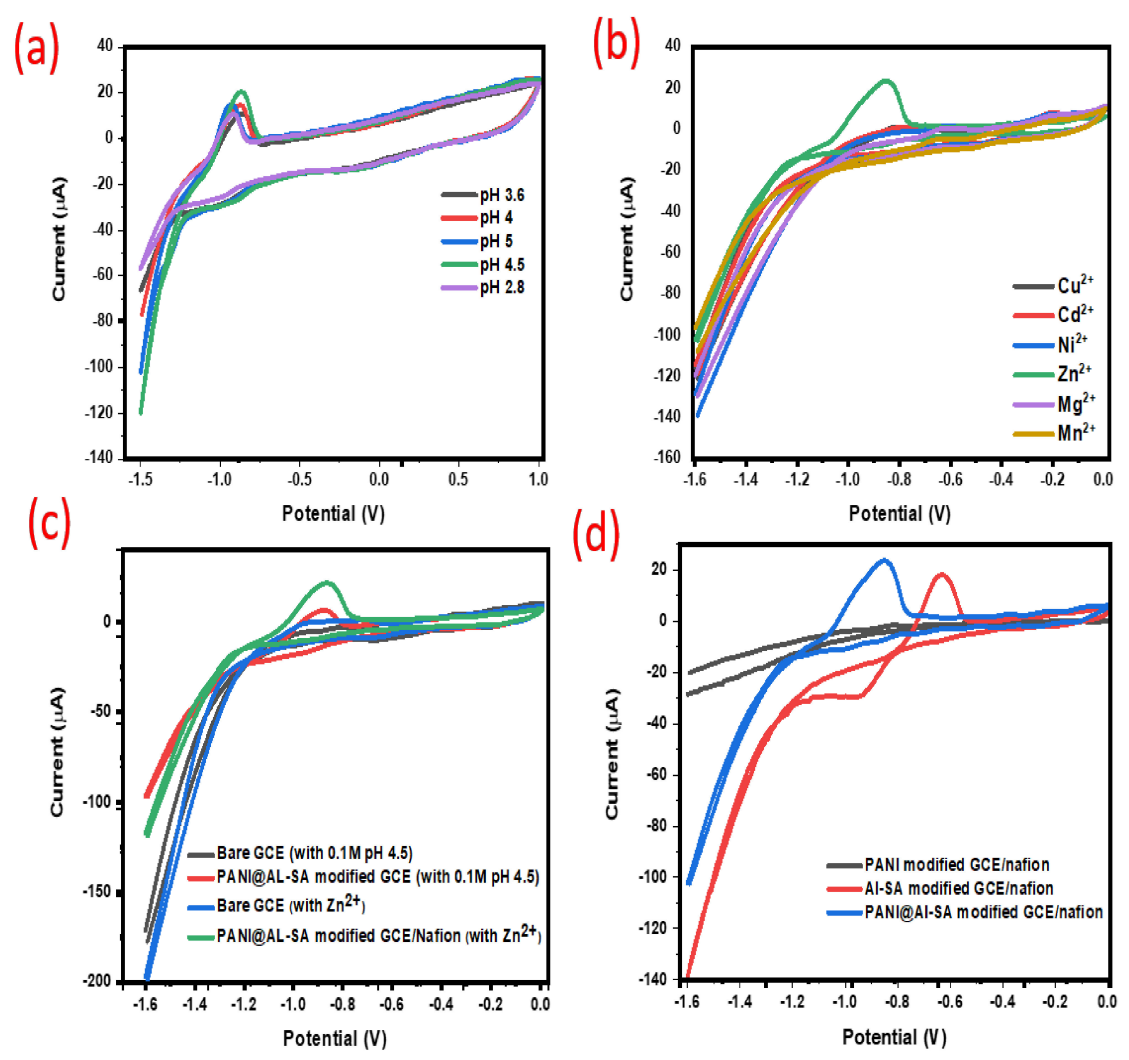
(**a**) CV response of 0.03 mM Zn^2+^ in pH solutions at a scan rate of 100 mV/s. (**b**) Comparative CV response of PANI@Al-SA/nafion modified GCE toward 0.03 mM of different metal ions in acetate buffer solution at pH 4.5 at a scan rate of 100 mV/s. (**c**) Cyclic voltammograms of response of bare and PANI@Al-SA/nafion modified GCE in the presence and absence of 0.03 mM Zn^2+^ in acetate buffer solution at pH 4.5 at a scan rate of 100 mV/s (**d**) Comparative CV response of PANI@Al-SA/nafion with its constituents in acetate buffer at pH 4.5 containing 0.03 mM of Zn^2+^ at scan rate of 100 mV/s.

The supporting electrolyte plays an important role in redox reactions on voltametric response. In order to estimate the effect of pH on the electrochemical behavior of PANI@Al-SA/nafion modified GCE toward 0.03 mM Zn^+2^, 0.1 M acetic acid (pH 2.8) and 0.1M acetate buffer solutions in the range 3.6 to 5 were studied. As it can be seen from Figure 7a, the anodic peak current increases with increasing pH until it reaches its maximum value at pH 4.5. Due to the effect of hydrogen dissolution in more acidic medium, the electrochemical response of zinc ion is favorable in low acidic medium. As a result, the pH 4.5 was employed as optimal pH in subsequent studies.

#### 3.3.2. Selectivity Study

To evaluate the performance behavior of PANI@Al-SA/nafion modified electrode as a sensor toward zinc ion, various metal ions were tested having the concentration of 0.03 mM in 0.1 M acetate buffer medium at pH 4.5. Figure 7b presents the selectivity study of PANI@Al-SA/nafion toward Cu^2+^, Cd^2+^, Ni^2+^, Zn^2+^, Mg^2+^ and Mn^2+^. It can be obviously noted the highest anodic peak current was toward Zn^2+^ as compared with other metal ions. This is clear indication the proposed cationic sensor is more selective toward zinc ion.

#### 3.3.3. Adjustment of Experimental Condition

In order to investigate the performance of PANI@Al-SA/nafion modified GCE toward the highly selective target analyte Zn^2+^, bare GCE, PANI@Al-SA/nafion modified GCE, PANI/nafion modified GCE and Al-SA/nafion modified GCE were evaluated using cyclic voltammetry approach in 0.1 M acetate buffer medium at pH 4.5 a supporting electrolyte.

The addition of 5% nafion to the PANI@Al-SA modified GCE surface improved the electrochemical response of sensor towards target ion because the role of nafion as ion- exchange film. Nafion containing the sulfonate groups which are negatively charged, therefore, at pH 4.5, the polymeric membrane behaves as a cation exchange membrane, facilitating non-faradaic preconcentration of metal cations [25].

From Figure 7c, a well-defined anodic peak potential at −0.86 V (ipa = 21 µA) was observed with PANI@Al-SA/nafion modified GCE while the bare GCE gave low peak current (i_pa_ = 6 µA). The resulting behavior attributed to the PANI@Al-SA nanocomposite improved electro-catalytic activity of the GCE. In addition, in 0.1 M acetate buffer of pH 4.5 (medium only in absence Zn^2+^ ion), the PANI@Al-SA/nafion modified GCE electrode displayed a fine cathodic peak as compared with bare GCE due to the existence of PANI@Al-SA on bare GCE surface Figure 7c.

In context, the electrochemical response of PANI@Al-SA/nafion modified GCE was examined in comparison with its constituents PANI and AL-SA in the presence of analyte zinc ion using 0.1 M acetate buffer medium at pH 4.5 as a supporting electrolyte. As displayed in Figure 7d, PANI@Al-SA/nafion modified GCE showed an anodic peak current at 23.5 µA (E_pa_ = −0.86 V) which is high current response as compared with the anodic peak current of Al-SA/nafion modified GCE at 18 µA ( E_pa_ = −0.63 V), while PANI/nafion modified GCE showed no response under the same conditions. These findings confirmed that the PANI@Al-SA/nafion/GCE electrode owns totally different physico-chemical properties from of its constituents PANI and Al-SA which is consistent with results of physiochemical characterization as discussed in the previous section.

#### 3.3.4. Effect of Scan Rate

**Figure 8:**
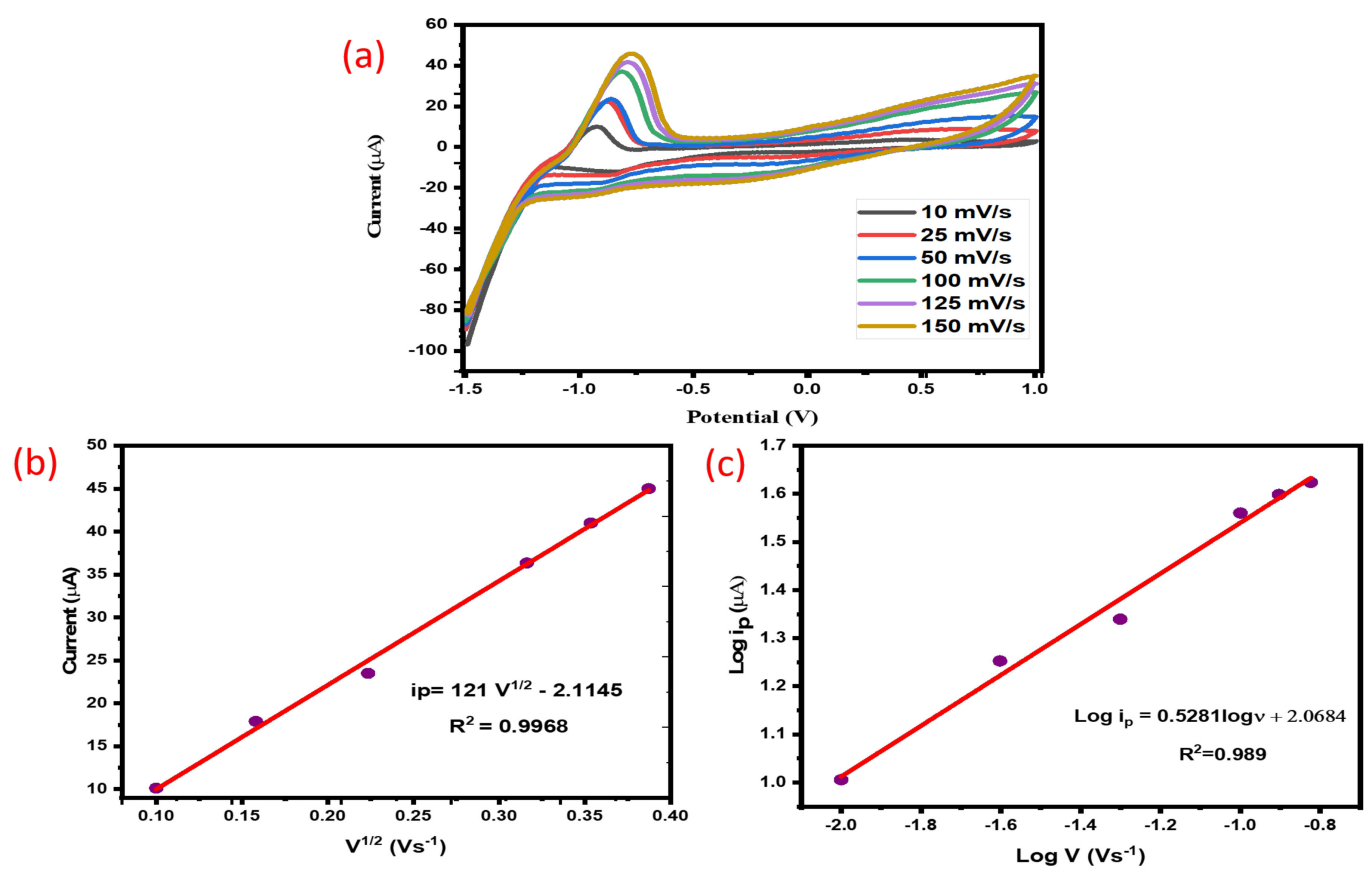
(**a**) Cyclic voltametric response of PANI@Al-SA/nafion modified GCE toward 0.065 mM Zn^2+^ at different scan rate. (**b**) Plot of current peak against square root of scan rate. (**c**) Plot of log of peak current against log scan rate.

Electrokinetic can well established thorough studying scan rate influence, which is significant for finding an equation that accurately represents the results. The CVs of PANI@Al-SA/nafion modified GCE in pH 4.5 and the concentration of Zn^2+^ 0.065 mM was carried out at various scan rate ranging 10–150 mv s^−1^ are depicted in Figure 8a. The result showed that as the scan rate increases, the peak current increases. Furthermore, a linear relationship was established between peak current and the square root of the scan rate as shown in Figure 8b with correlation coefficient (R^2^ = 0.99), demonstrating the process of the electrode reaction is a diffusion-controlled. It can be emphasizing this result by plotting the logarithm of anodic peak current (Log ip) versus the logarithm of scan rate (Log v) as illustrated in Figure 8c, with the regression equation was found:

$$\log i_{p}=0.5281\;logv+2.0684\;\;\;\;\;\;\;\;\;\;\;\;\;\;\;\;\;\;\;R^{2}=0.989\;$$

The slope value of electrode reaction of zinc ion at PANI@Al-SA/nafion modified GCE was found to be 0.52, confirming a highly favorable the electrode reaction of Zn^2+^ ion is considered a diffusion-controlled process, which is near to the theoretical value of 0.5 for a diffusion-controlled process [26,27].

Meanwhile, the parameters which related to the totally irreversible diffusion-controlled like electron transfer coefficient (α) and number of electrons transferred in this process were calculated by employing the following equations [28,29,30,31]:

(6) $$\alpha =47.7/\left(E_{p}-E_{p/2}\right)$$

(7) $$\mathrm{Ep}=\left(\frac{b}{2}\right)\mathrm{logv}+\mathrm{constant}$$

(8) $$b=\frac{2.303\mathrm{RT}}{\left(1-\alpha \right)\mathrm{nF}}$$

where, v refer to the scan rate, R denotes the gas constant 8.314 J.K^−1^mol^−1^, F is Faraday’s constant 96500 C/mol and b is the Tafel slope resulting from the slope of graph in Figure S3. Other parameters have their usual meaning. Therefore, the α and n were found to be 0.86 and 1.96 ≈ 2, respectively (ESI: S5).

Also, the diffusion coefficient for zinc ion was evaluated by applying the Equation (5) and the slop of the linear region of the peak current, ip (A) against square root of scan rate, $\sqrt{v}$ (V/s) in 0.065 mM Zn (II) in 0.1 M acetate buffer medium of pH 4.5 (Figure S4). The calculated diffusion coefficient for zinc ion is 7.96 × 10^−6^ cm^2^ s^−^^1^, which close the value obtained in literature [32].

#### 3.3.5. Quantitative Electrochemical Determination of Zinc Ion by Linear Sweep Voltammetry (LSV)

**Figure 9:**
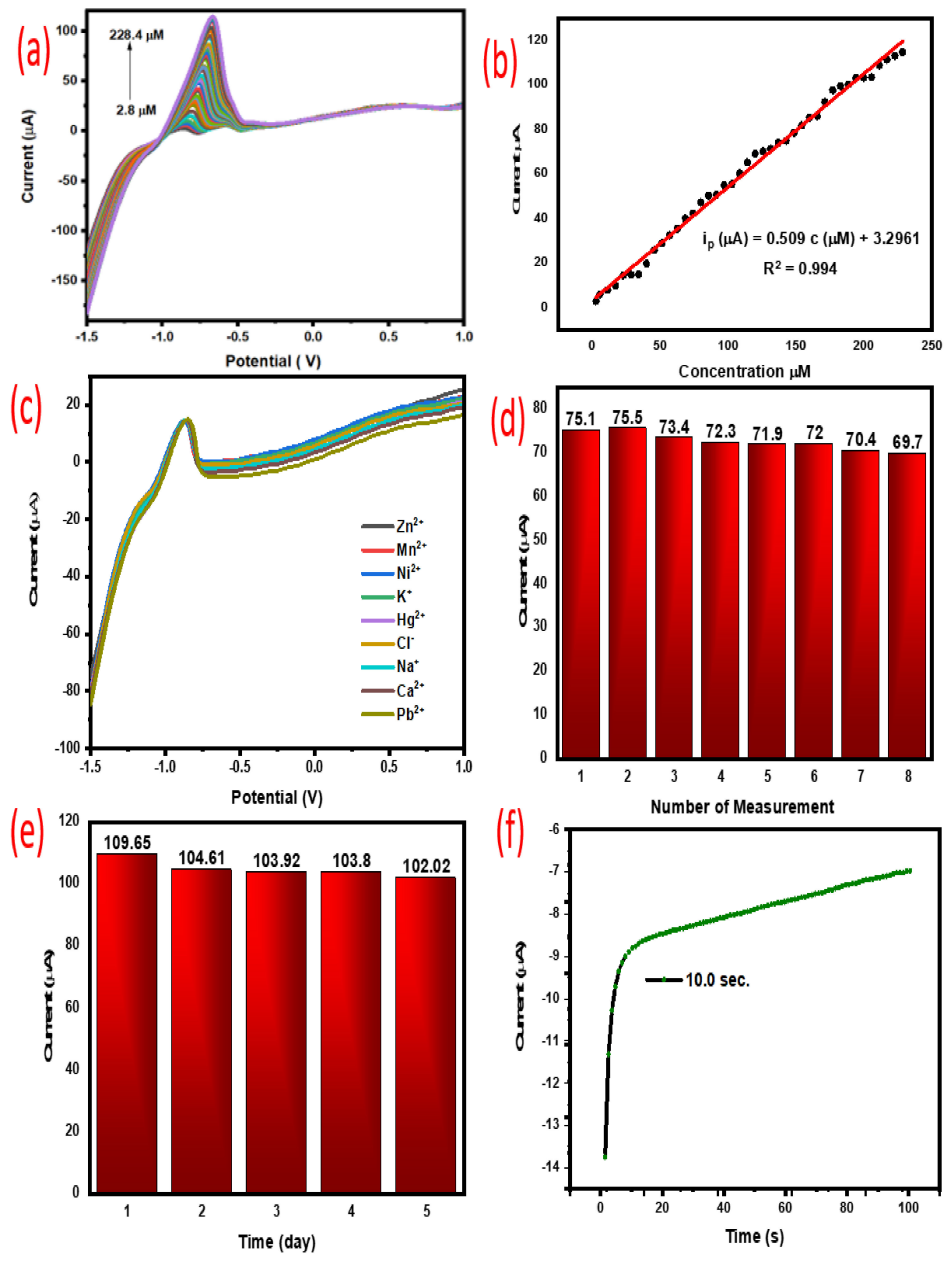
(**a**) LSV response of PANI@Al-SA/nafion modified GCE with different concentrations of Zn^2+^ at a scan rate of 100 mV/s. (**b**) Calibration plot the peak current against Zn^2+^ concentration. (**c**) Voltammetric response of PANI@Al-SA/nafion modified GCE in 0.02 mM Zn^2+^ with addition of interferents (2-fold concentration) at scan rate 100 mV/s. (**d**) Given peak currents of PANI@Al-SA/nafion modified GCE in 0.14 mM Zn^2+^ recorded at eight consecutive measurements. (**e**) obtained current response of PANI@Al-SA/nafion modified GCE in 0.2 mM Zn^2+^ for long period (5 days). (**f**) estimation of response time for detection of zinc ion.

Linear sweep voltammetry (LSV) technique was employed for quantitative determination of zinc ion in the present study because LSV is high sensitive and precise particularly for an irreversible reaction process. The electrochemical behavior of Zn^2+^ ion with different concentration on conducting PANI@Al-SA/nafion modified GCE was shown in Figure 9a. The peak current was increased linearly in concentration range of 2.8–228.4 µM. Based on the calibration curve Figure 9b, it was covered a wide range with a good correlation coefficient (R^2^ = 0.994) between the concentration and peak current. In addition, the analytical figures of merit for zinc ion detection by PANI@Al-SA/nafion modified GCE were determined in terms of limit of detection (0.59 µM), limit of quantification (1.96 µM) and sensitivity (7.14 µA µM^−1^ cm^−2^), at signal-to-noise ratio of three in this experiment (3N/S). The proposed sensor PANI@Al-SA/nafion modified GCE was compared with the previously reported modified electrodes for Zn^2+^ions detection and summarized in Table 1.

PANI@Al-SA/nafion modified GCE showed a large linear dynamic range (LDR) with a good detection of limit (LOD).

#### 3.3.6. Interference Study

The effect of coexisting ions on the peak current of PANI@Al-SA/nafion modified GCE toward Zn^2+^ was studied to estimate the applicability of the proposed sensor. The peak current response of 0.02 mM Zn^2+^ and 2-fold of concentration of matrix ions like Mn^2+^, Ni^2+^, Hg^2+^, Ca^2+^, pb^2+^, Cd^2+^, Cu^2+^, Na^+^, K^+^, Cl^-^ and OH^-^ under optimized condition shown in Figure 9c. Result displayed that the peak current of Zn^2+^ did not significantly interfere in presence of interfering ions with acceptable relative standard deviation (RSD) of the current response 3.27%.

#### 3.3.7. Stability of the Proposed Sensor

The stability of peak current of PANI@Al-SA/nafion modified GCE toward Zn^2+^ was examined in terms of repeatability or (test-retest reliability) and reproducibility. Analyses of these terms were associated to the precision and accuracy of repeated analyses in the identical conditions. Repeatability test was determined by taking the peak current in eight repetitive measurements in 0.14 mM Zn^2+^ at acetate buffer medium of pH 4.5 under the identical condition as shown in Figure 9d. The relative standard deviation (RSD) of the eight repetitive peak currents was 2.83% which indicate to the current response was well repeatable.

The reproducibility test of the peak current of PANI@Al-SA/nafion modified GCE toward Zn^2+^ was performed for a long period of around following five days under the identical condition as presented in Figure 9e. Result was evaluated in terms of RSD (2.74%) which shows the proposed sensor has a good stability through long period of time.

In addition, the velocity of the sensor’s response was estimated by recording the current (µA) against time (s) as displayed in Figure 9f. The response time of PANI@Al-SA/nafion modified GCE toward Zn^2+^ around 10 s. This finding suggested that the PANI@Al-SA/nafion modified GCE based sensor is fast response towards detection of zinc ion.

#### 3.3.8. Application of the Proposed Sensor in Real Samples

In order to estimate the reliability of the analytical developed method, it was implemented to quantification of zinc ions in real samples. Results demonstrate the good recoveries was obtained in application field as displayed in Table 2, suggesting that the applicability of the proposed sensor for the determination of Zn^2+^ ion in real samples.

## 4. Conclusions

In this study, polyaniline@aluminum–succinate MOF, a novel electrically conducting nanocomposite was prepared by incorporation of aluminum succinate MOF into the conducting polyaniline. The prepared nanocomposite was characterized by various techniques to confirm successful preparation. DC electrical conducting property was determined by four probe method which showed the PANI@MOFs composite as semiconducting. PANI@Al-SA was used for the preparation of modified GCE (PANI@Al-SA/nafion/GCE) for electrochemical studies for the Zn (II) ion detection in aqueous medium. The electrochemical sensor exhibited high selectivity towards Zn(II) ion. The performance of PANI@Al-SA demonstrated a wide linear dynamic range, good repeatability, short response time and appropriateness for real environmental sample analysis. Hence, the present study is a promising and reliable method for zinc ion determination in real samples for public health and safety.

## Figures and Tables

**Table 1:** Comparison of sensor performances for detection of Zn^2+^ with different modified electrodes by electrochemical methods.

Modified Electrode	Technique	LR(µM)	LOD (µM)	Ref
Hg thin film SPE	DPASV	Up to 15.29	0.85	[33]
Bismuth oxide modified ink SPE	SWASV	0.6–2.29	0.45	[34]
Microfabricated bismuth electrodes	ASV	5–50	6	[35]
Graphite felt electrode	LSSV	1–100	0.5	[36]
PANI@Al-SA /nafion modified GCE	LSV	2.8–228.6	0.5	This study

SPE: Screen-printed electrode; DPASV: Differential pulse anodic stripping voltammetry; SWASV: Square wave anodic stripping voltammetry; LSSV: Linear sweep stripping voltammetry.

**Table 2:** Data obtained for the determination of zinc ions in real samples using the proposed (LSV) method.

Sample	Spiked Concentration (µM)	Found Concentration (µM)	RSD (%) (*n* = 3)	Bias	Recovery (%)
Tap water	0	0	-	-	-
	3	2.94	1.4	−0.06	98.06
	6	5.8	1	−0.23	96.11
Bottled water	0	0	-	-	-
	3	2.91	1.45	−0.08	97.22
	6	5.86	0.8	−0.13	97.78

## Supplementary Materials

The following are available online at https://www.mdpi.com/article/10.3390/polym13193383/s1, Figure S1(a): (a) Cyclic voltametric response of PANI@Al-SA modified GCE containing 1mM ferricyanide (in 0.1M KC) at a scan rate from 5 mV/s to 50 mV/s Inset: The pink, green and orang arrows indicate scan direction, anodic and cathodic peaks, respectively. Figure S1(b): Plot of anodic peak current against square root of scan rate. Figure S2: (a) Graphs showing equivalent circuits used for Electrochemical impedance spectroscopy fitting for bare GCE and (b) PANI@Al-SA modified GCE; Rct: charge transfer resistance, Rs: solution resistance, CPE: constant phase element and W:Warburg impedance. Figure S3: Tafel plot obtained from CV in Figure 9(a). Figure S4: plot of current peak vs square root of scan rate which obtained from CV in Figure 9(a); Table S1: Result of EIS data fitting for bare GCE. Table S2: Result of EIS data fitting for PANI@Al-SA modified GCE.
