# Supplementary material for: Efficient Synthesis and Characterization of Polyaniline@Aluminium–Succinate Metal-Organic Frameworks Nanocomposite and Its Application for Zn(II) Ion Sensing
Source: Polymers (Basel). 2021 Sep 30;13(19):3383. doi: 10.3390/polym13193383 (PMC8512637; doi:10.3390/polym13193383)
Supplement: Supplementary file 1 [file polymers-13-03383-s001.zip › polymers-1362191-supplementary.pdf]

Efficient Electrocatalytic Performance of PANI@Al-SA MOF Nanocomposite for Zn(II) Reduction

Amjad E. Alsafrani [a,b], Waheed A. Adeosun[a], Hadi M. Marwani[a,c], Imran Khan[d], Abdullah M. Asiri[a,c], Anish Khan[a,c*]

[a] Department of Chemistry, Faculty of Science, King Abdulaziz University, P.O Box 80203, Jeddah 21589, Saudi Arabia.

[b] University of Jeddah, College of science, Department of Chemistry, Jeddah, Saudi Arabia.

[c] Center of Excellence for Advanced Materials Research, King Abdulaziz University, P.O Box 80203, Jeddah 21589, Saudi Arabia.

[d] Applied Sciences and Humanities Section, University Polytechnic, Faculty of Engineering and Technology, Aligarh Muslim University, Aligarh – 202002, India

SUPOORTING INFORMATION TO THIS ARTICLE

S1 (a) Cyclic voltametric studies of PANI@Al-SA modified GCE toward 1mM ferricyanide at different scan rates.

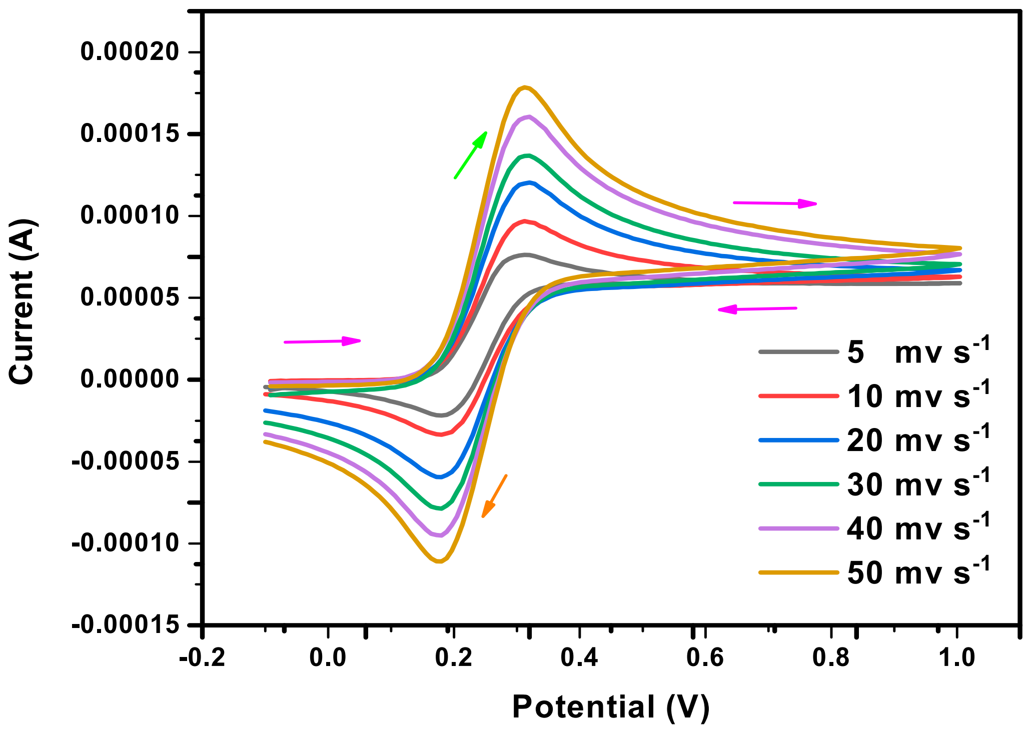

Figure S1. (a) Cyclic voltametric response of PANI@Al-SA modified GCE containing 1mM ferricyanide (in 0.1M KC) at a scan rate from 5 mV/s to 50 mV/s Inset: The pink, green and orang arrows indicate scan direction, anodic and cathodic peaks, respectively.

S1 (b) Calibration plot of data obtained from cyclic voltametric of PANI@Al-SA modified GCE toward 1mM ferricyanide at various scan rates.

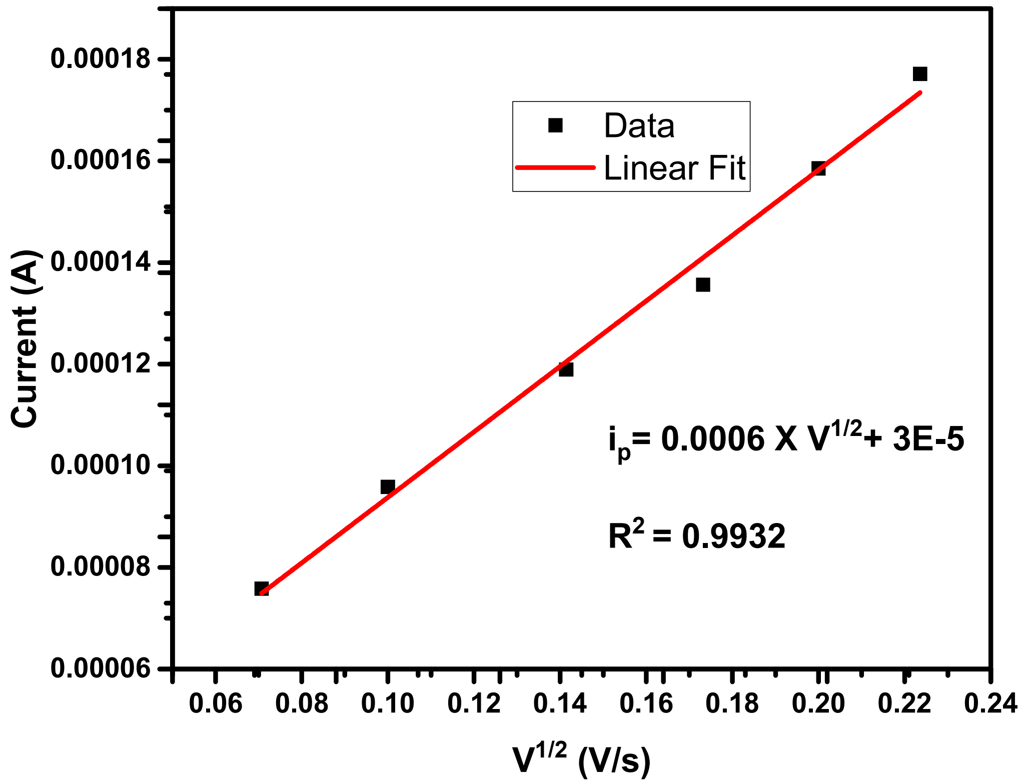

Figure S1. (b) Plot of anodic peak current against square root of scan rate.

S2 Equivalent circuits applied for Electrochemical impedance spectroscopy for bare glassy carbon electrode (GCE) and PANI@Al-SA coated GCE.

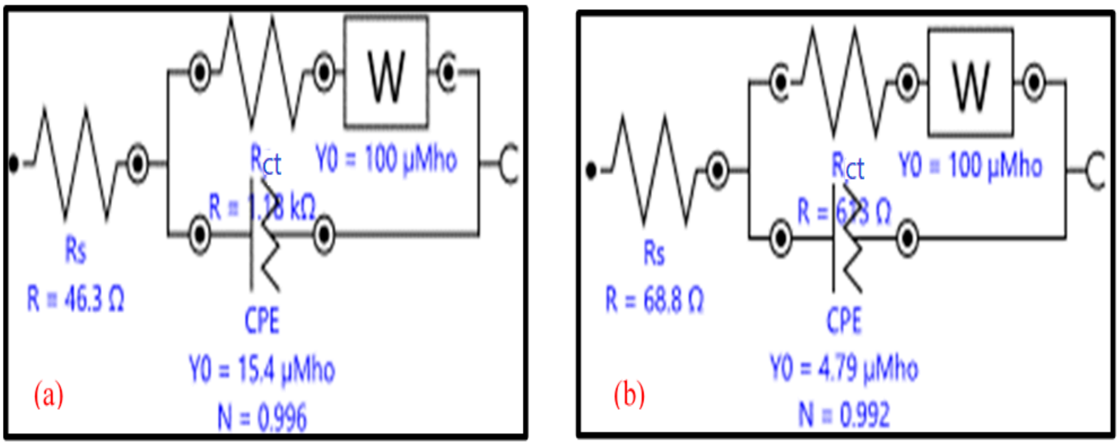

Figure S2. (a) Graphs showing equivalent circuits used for Electrochemical impedance spectroscopy fitting for bare GCE and (b) PANI@Al-SA modified GCE; Rct: charge transfer resistance, Rs: solution resistance, CPE : constant phase element and W:Warburg impedance.

S3 Tafel plot obtained from cyclic voltametric of PANI@Al-SA coated GCE toward 0.065 mM zinc ion at different scan rate.

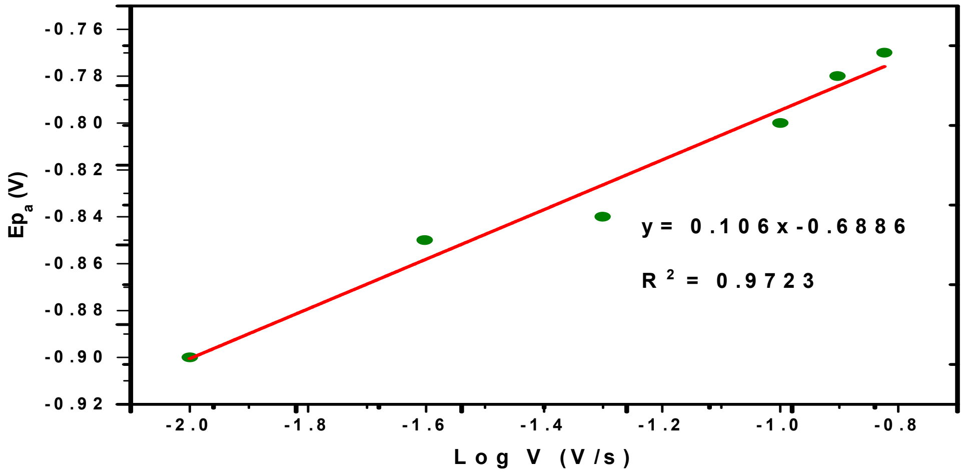

Figure S3. Tafel plot obtained from CV in Fig. 9 (a)

S4 **Calibration plot of data obtained from cyclic voltametric of PANI@Al-SA modified GCE toward 0.065 Zn^{2+} at scan rate from 10 mv/s to 150 mv/s**

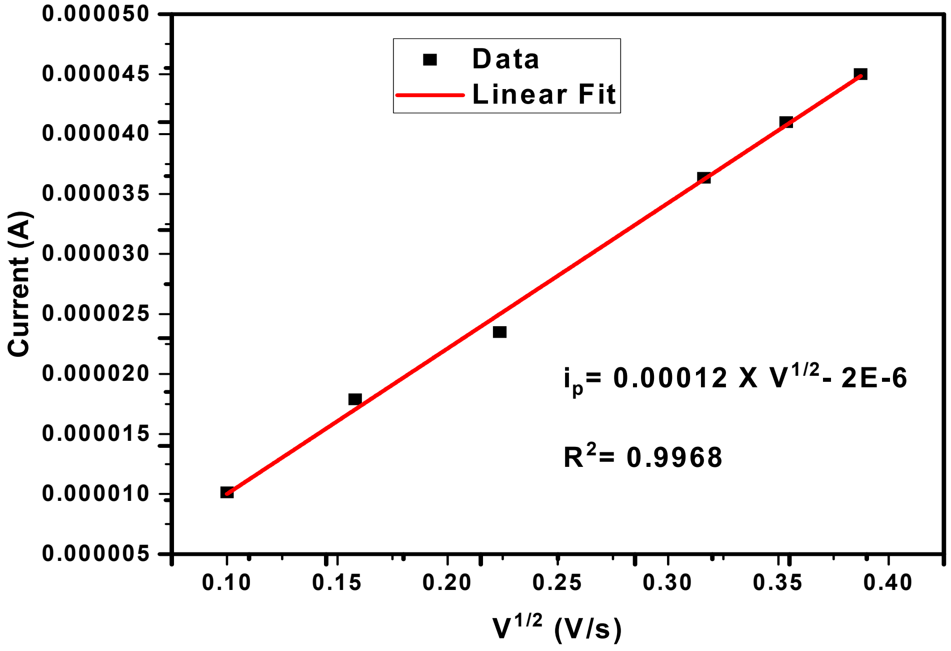

Fig. S4. plot of current peak vs square root of scan rate which obtained from CV in Fig. 9

(a)

Table S1. Result of EIS data fitting for bare GCE.

Element	Parameter	Value
Rs	R	46.31
Rct	R	1183.3
CPE	Y0	1.5397E-05
	N	0.99617
W1	Y0	0.0001

Table S2. Result of EIS data fitting for PANI@Al-SA modified GCE

Element	Parameter	Value
Rs	R	68.795
Rct	R	612.82
CPE	Y0	4.7935E-06
	N	0.99203
W1	Y0	0.0001

S5 Electrochemical variables calculations

Electron transfer coefficient (α)

$\alpha = 47.7/(E_p - E_{p/2})$

α= 47.7 / [(-922) – (-977)] mV

α= 0.86

Number of electrons transferred (n)

n calculated from combination Tafel plot with the following eq:

$$b = \frac{2.303RT}{(1-\alpha)nF}$$

n= 1.96

Diffusion coefficient (D)

D was calculated by using Randles sevcik equation

$i_p = (2.69 \times 10^5) \times A \times n^{3/2} \times D^{1/2} \times C \times v^{1/2}$

$D = 7.96 \times 10^{-6}\ cm^2\ s^{-1}$
